# Reach and effectiveness of a worksite health promotion program combining a preventive medical examination with motivational interviewing; a quasi-experimental study among workers in low socioeconomic position

**DOI:** 10.1186/s12889-023-16908-w

**Published:** 2023-10-31

**Authors:** David van de Ven, Merel Schuring, Tessa A. Kouwenhoven-Pasmooij, Philip Blom, Alex Burdorf, Suzan J. W. Robroek

**Affiliations:** 1 Department of Public Health, Erasmus University Medical Center, P.O. Box 2040, 3000 CA Rotterdam, the Netherlands; 2 VitAll, Rotterdam, the Netherlands; 3 Health Centre Zwolle, Zwolle, the Netherlands

**Keywords:** Workplace, Health promotion, Motivational interviewing, Socioeconomic position, Body mass index, Health behaviour, Propensity score

## Abstract

**Background:**

This study aimed to evaluate individual characteristics associated with participation and effectiveness of a worksite health promotion program with motivational interviewing targeting health and health behaviour among Dutch workers in low socioeconomic position.

**Methods:**

In a production company and a hospital, 838 workers were invited for a Preventive Medical Examination and subsequent coaching with motivational interviewing up to 7 sessions within 6 months. Follow-up information was collected after 6 months. Characteristics associated with participation in coaching were assessed with logistic regression models. The effectiveness of coaching on body mass index (BMI), bodyweight, self-rated health, vigorous physical activity, smoking, alcohol intake, fruit- and vegetable consumption, work ability, and sickness absence was evaluated with linear regression models and on participation in health promotion activities with logistic regression analysis. The analyses on effectiveness were performed without and with propensity score adjustment.

**Results:**

Of the 838 invited workers, 313 workers participated in the Preventive Medical Examination and follow-up data were available for 176 workers, of whom 100 workers with increased cardiovascular risk attended coaching. The majority of workers with obesity (73%), overweight (60%), and unhealthy behaviours (58%-69%) at baseline participated in motivational interviewing. Males, workers with overweight or obesity, workers at the production company, workers with insufficient vigorous physical activity, and workers with a low educational level were most likely to participate in coaching. Coaching with motivational interviewing after the Preventive Medical Examination was associated with a 4.74 times higher likelihood [95% confidence interval (CI): 1.99;11.32] to participate in health promotion activities and 10.9% (95%CI: 0.6;21.3) more persons who quit smoking compared to workers without coaching. No statistically significant effects were observed on BMI, bodyweight, health, health behaviour, work ability and sickness absence.

**Conclusions:**

The program combining a Preventive Medical Examination with follow-up coaching reached – as intended – workers with obesity or overweight, those with a low education and with unhealthy behaviours. Adding coaching with motivational interviewing to a Preventive Medical Examination contributed to higher participation in health promotion activities and an increase in smoking cessation after 6 months among workers with a lower socioeconomic position, but was not effective on other outcomes.

**Trial registration:**

The study was registered retrospectively in the Netherlands Trial Register as NL8178 on 22/11/2019.

**Supplementary Information:**

The online version contains supplementary material available at 10.1186/s12889-023-16908-w.

## Background

Obesity and unhealthy behaviour (i.e. lack of physical activity, smoking, alcohol consumption and poor nutrition) are important modifiable risk factors for cardiovascular diseases [1, 2]. In addition, these factors increase the risk of higher sickness absence [3] and lower work ability [4, 5]. Obesity and unhealthy behaviour are more prevalent among workers in lower socioeconomic position [6]. Decades of research on the effectiveness of workplace health promotion programs (WHPPs) have shown small improvements in health and health behaviour for workers in general [7], and specifically for workers in low socioeconomic position [8]. In order to decrease socioeconomic health inequalities among workers, a need of effective WHPPs exist which are tailored to workers in low socioeconomic position. Participation in health promotion is generally low, also for workers in lower socioeconomic position [9]. In order to develop strategies to tailor WHPPs to this group of workers who need it the most, insight is needed in the factors that contribute most to participation among this group.

Health-risk assessment is widely considered as an effective approach to facilitate participation in other health promotion activities [10]. However, to further promote behavioural change, health risk assessments should be complemented with other intervention components. Motivation is an important driver of behavioural change. According to the self-determination theory, behaviour is guided by intrinsic motivation or the internalization of initially non-intrinsic motivation [11, 12]. In addition, this theory states that persons’ needs for competence regarding their ability to change, for autonomous rather than forced action, and for relatedness are the basis for self-motivated behavioural change. Motivational interviewing (MI) is viewed as a promising method to increase self-motivated behavioural change. This method can be defined as a collaborative coaching style aimed to strengthen self-motivation by evoking reasons to change rather than reasons to retain the current behaviour [13]. This includes techniques such as asking open-ended questions, reflective listening, and giving confirmation. It is assumed that when persons have both reasons to change as well as to retain their current behaviour a directive coaching style characterized by giving advice and persuasion results in resistance and a higher likelihood that persons argue against change. This assumption is supported by studies on the effectiveness of MI, which show higher effectiveness of MI in preventing unhealthy behaviour in a variety of health- and social care settings when compared with usual care or directive brief advice [14]. Scarce evidence also shows that MI improves health behaviour in lower socioeconomic groups [15, 16].

Although Kouwenhoven-Pasmooij et al. [17] indicated that adding motivational interviewing to a web-based health risk assessment with advice reduced bodyweight among workers in the military, police and hospital, it is unclear yet whether these components in a WHPP contribute to increased effectiveness specifically among workers in lower socioeconomic position. In addition, it is unclear yet which characteristics contribute most to reach of workers in lower socioeconomic position in WHPPs in general and MI coaching in particular. Therefore, an existing Dutch WHPP [17] was optimized specifically for this group of workers. The current program is primarily focussed on improving health and health behaviour. It is hypothesized that more healthy behaviour contributes to an increased ability to cope with the demands at work and may therefore increase work ability. The aims of this study were to investigate 1) which individual characteristics were associated with participation in motivational interviewing, and 2) the extent to which the WHPP was effective in improving health, health behaviour, work ability and sickness after 6 months among Dutch workers in lower socioeconomic position in a hospital and production company.

## Methods

### Study design and population

The WHPP was evaluated by employing a quasi-experimental study carried out at 14 departments in a hospital and a production company in the Netherlands. Workers in lower paid jobs who often performed physically strenuous work were targeted. These workers included kitchen workers, telephone operators, nutrition assistants, logistic staff, mechanics, paint sprayer, production workers. Eligibility criteria for individual workers in the study included 1) being in paid employment and 2) working at least 12 h a week. Participants were included between June 2019 and March 2020 after providing informed consent. At baseline a web-based questionnaire with questions on health, health behaviour, work ability, sickness absence, working conditions, and demographics was administered, and anthropometric measurements were carried out as part of the preventive medical examination. Six months after the baseline measurements participants were asked to fill-out a second web-based questionnaire with questions on the primary and secondary outcome measures and additional questions on participation in intervention activities. The Medical Ethics Review Committee of the Erasmus University Medical Center in Rotterdam waived the requirement for formal ethical application as the Medical Research Involving Human Subjects Act did not apply for the current study (MEC2018-1717). Informed consent was obtained from all study participants. The study is registered in the Netherlands Trial Register as NL8178 on 22/11/2019 (https://trialsearch.who.int/Trial2.aspx?TrialID=NL8178).

### The worksite health promotion program

Within the hospital the study was announced through e-mail and in the production company workers were notified about the study via their supervisor. The intervention consisted of the following components:

1. Preventive Medical Examination (PME), consisting of a web-based questionnaire with questions on health, health behaviour, work ability, sickness absence, working conditions, and anthropometric measurements.
2. Tailored coaching based on MI, up to 7 sessions within 6 months.

All workers enrolled voluntarily in the study by completing the PME, which took approximately 45 min. The communication and wording of the questions in the web-based questionnaire was adapted to the language level of the workers. Workers were given the opportunity by their supervisors to participate in the PME during working hours. In the hospital, the questionnaire was self-administered and workers performed their own anthropometric measurements with a toolbox they received at home. In order to improve accessibility of the PME at the hospital, workers could perform their measurements at the occupational health service. In addition, computers were made available at the department to fill out the questionnaire. At the production company, PMEs were already frequently offered to workers before the current program. The current PME was integrated in their existing procedure. In accordance to their existing PME procedure, the company doctor's assistant administered the questionnaire and performed the anthropometric measurements face-to-face. After completing the PME, participants received a computer-generated overview of their results on specific factors related to health, health behaviour and work, and their cardiovascular risk profile. Results were represented as no risk (green), low risk (orange), and high risk (red). Focus groups that were performed at the organizations as part of the development of the program indicated that when the results were presented this way the presence and magnitude of the health risks were conveyed well and that they would be more inclined to change their behaviour.

Participants with a low or high cardiovascular risk were given the opportunity to receive tailored face-to-face coaching based on MI (30 min per session). These coaching sessions were focused on change in physical activity, smoking, alcohol consumption, nutrition, or relaxation, depending on the goal setting of the workers. In the hospital, workers with a low cardiovascular risk could participate in a maximum of 2 coaching session with a lifestyle coach and those with a high risk could receive 1 coaching session with an occupational physician (OP) and a at least 1 session with a lifestyle coach. Workers with a low risk at the production company could participate in at least 1 coaching session with either a dietician, physical therapist, or social worker, and workers with a high cardiovascular risk could receive 1 session with an OP and at least 1 with the other professionals. These MI sessions had to take place between the PME and follow-up measurement after 6 months. Whereas coaches within the production company received 3 days of training in MI, coaches within the hospital received 2 days of training in MI. Most of the coaches in the hospital already completed a training or workshop in MI before development of the program.

When participants had the intention to change their behaviour and were willing to undertake actions during the coaching sessions, they received suggestions on health promotion activities based on their personal preference. Participants could either independently undertake action to change their behaviour or they could take part in health promotion activities offered by the organisations. The latter activities include discount on gym membership, (e-health) interventions for quitting smoking, improving vitality, and mindfulness, and the use of mobile apps to track and improve physical activity. Reach was defined as participation in the MI after the PME compared to persons only participating in the PME.

### Measurements

#### Primary outcome measures

##### Participation in health promotion activities

Participation in health promotion activities was measured after 6 months using the question ‘Since you participated in the PME 6 months ago, have you taken action in order to improve your health behaviour (physical activity, smoking, alcohol use, nutrition or relaxation). Workers could indicate whether they individually attempted to improve their health behaviour or participated in health promotion activities offered by the organisation (e.g. courses on smoking cessation, mindfulness or vitality, or discount on gym membership). If workers indicated that they had taken action on at least one health behaviour, this was considered as having participated in health promotion activities.

##### Bodyweight and BMI

During the PME at baseline bodyweight and height were measured. Whereas workers in the hospital measured their own bodyweight and height, at the production company these measurements were performed by the doctor’s assistant. After 6 months self-reported data on bodyweight were collected. BMI was derived from the weight and baseline height of participants and expressed in kg/m^2^. BMI was categorised into workers with a healthy weight (18.5 kg/m^2^ to 25 kg/m^2^), overweight (25 kg/m^2^ to 30 kg/m^2^) workers and obese workers (≥ 30 kg/m^2^).

#### Secondary outcome measures

##### Health behaviour and self-rated health

At baseline and after 6 months respondents were asked to indicate their current health behaviour with respect to the number of days a week (0–7 days) they performed vigorous physical activity (activities which cause persons to sweat) during leisure time, smoking status (yes/no), alcohol consumption in alcohol-units per week (‘1 = less than 1 glass per week’ to ‘6 = more than 28 glasses per week’), daily intake of fruit (‘1 = none’ to ‘6 = 3 or more pieces of fruit a day’) and vegetable consumption (‘1 = none’ to ‘6 = 4 or more spoons a day’). Dichotomous variables were made based on adherence to Dutch guidelines (yes/no) for performing leisure time vigorous physical activity for at least 2 days a week [18], not smoking, not drinking more than 7 glasses of alcohol per week [19], eating at least 2 pieces of fruit a day, and at least 4 servings of vegetables (200 g) per day [19]. Self-rated health was measured at baseline and after 6 months by asking respondents to rate their generate health on a scale from 1 (very good) to 5 (very bad) [20]. Self-rated health was categorized into workers having good or very good self-rated health, and those having less than good self-rated health.

##### Work ability and sickness absence

Self-reported work ability was measured at baseline and after 6 months using the first dimension of the Work Ability Index (WAI) [21]. Respondents were asked to indicate their current work ability as compared to their lifetime best on a scale from 0 (not able to work) and 10 (lifetime best). At baseline and after 6 months it was examined how many days in the past year participants were not able to work due to sickness, admission to hospital or research on a 5-point scale (‘1 = 0 days’ to ‘5 = 100 to 365 days’). Dichotomous variables were made for work ability (work ability higher than 6 versus 6 or lower) and sickness absence (less than 10 days sickness absence versus at least 10 days).

#### Covariates

At baseline, socio-demographic information was collected on gender, age in years, highest completed educational level, and marital status. Age was categorized in 4 groups; lower than 30 years, between 30 and 40 years, between 40 and 50 years, and 50 years and older. Education was divided in three groups; low (no education, primary school, lower and intermediate secondary schooling, or lower vocational schooling), intermediate (higher secondary schooling, or intermediate vocational schooling), or high education (higher vocational schooling, or university). For marital status being married or cohabiting was compared with unmarried, divorced or single workers. Additionally, experience of financial pressure at baseline was assessed and dichotomized (intermediate or high financial pressure versus little or no financial pressure). Several working conditions were assessed at baseline. Autonomy (Cronbach’s alpha = 0.81) and work pressure (Cronbach’s alpha = 0.67) each were measured with 3 items derived from the Job Content Questionnaire (JCQ) with the answer categories 1 (always), 2 (often), 3 (regularly), 4 (sometimes), and 5 (never) [22]. Based on mean scale scores, low autonomy (scores 4 to 5) was compared with high autonomy (scores 1 to 3) and high work pressure (scores 1 to 3) with low work pressure (scores 4 to 5). Dichotomous variables (yes/no) were made for other work-related aspects including having a high physical workload (whether or not workers had to lift, push, pull or turn heavy loads) during daily work, performing shift work, performing mainly physically demanding tasks or both mentally and physically demanding tasks, less than good work-life balance, and working at least 36 h a week.

### Delivery of the intervention

Intervention exposure was indicated by the number of MI coaching sessions workers attended and the mean total duration of these sessions as reported by coaches. The number of health promotion activities workers participated in was acquired from the 6 month follow-up questionnaire. The quality of MI coaching was determined by audio recordings, which were made by the coaches. The OPs and dietician were instructed to record every 4^th^ session they provided and the other professionals had to record every 3^rd^ session according to an agreed protocol, to ensure random selection of the recordings. The frequency of recoding was set lower for the OPs and dietician as they were expected to meet more participants than the other professionals. Recorded sessions were analysed using the Motivational Interviewing Treatment Integrity code (MITI) version 4.2.1 [23], which represents a reliable instrument for assessing MI treatment integrity [24]. Coding was done by a trained researcher (DV). Quality of MI was expressed by the mean technical (average of global scores cultivating change talk and softening sustain talk)- and relational score (average of global scores partnership and empathy), both on a scale from 1 to 5. A technical score of 3 and relational score of 4 was indicative of acceptable quality. The proportion of recordings with at least 40% complex reflections and ratio of reflections versus questions ≥ 1 was considered as adequate MI. In addition, self-reported data from the 6-month follow-up questionnaire was used to indicate the percentage of workers (strongly) agreeing with the following statements on the contact with coaches; ‘I was treated in a pleasant way’, ‘the coach had expertise’, ‘my medical data was treated confidentially’, ‘I was satisfied with the contact in general’, ‘the contact lived up to my expectations’, ‘because of the contacts I know how to improve my health behaviour’, ‘the contact with the coach contributed to change in health behaviour’, and ‘the number of contact moments was sufficient’. Answers were given on a 5-point scale ranging from 1 (strongly disagree) to 5 (strongly agree).

### Statistical analysis

Baseline characteristics were compared between 1) workers who completed the PME and attended subsequent MI coaching sessions, and 2) workers who completed the PME but did not attend MI coaching using chi-square tests. Logistic regression analysis was performed to determine whether baseline characteristics were associated with drop out after 6 months.

Associations between baseline characteristics and participation in the PME and follow-up MI coaching sessions were tested with multivariate logistic regression analyses. Backward elimination based on maximum likelihood estimates was used to select a combination of characteristics that contributed most to participation in the intervention group. For each individual, the propensity score was estimated, indicating the probability to participate in the PME as well as MI coaching compared to participation in the PME without additional MI coaching.

Multivariate logistic regression analysis was performed to test whether MI sessions were associated with participation in health promotion activities. This analysis was adjusted for sociodemographic characteristics (gender, age, educational level), organisation, and the propensity score to decrease the impact of selection bias and the voluntary choice to participate in the intervention due to the quasi-experimental study design [25]. Changes in health, health behaviour, work ability and sickness absence between baseline and 6 months within each group were evaluated using paired T-tests for continuous variables and McNemar’s test for dichotomous variables. The effectiveness of subsequent MI coaching after 6 months compared to workers who did not attend MI coaching was analysed with linear regression models. Analyses were performed among workers with complete data at baseline and 6 months follow-up. First, we employed models in which participation in MI coaching was included as independent variable, with adjustment for baseline values on outcome measurements, socio-demographic characteristics (gender, age, educational level), and organisation. Next, the propensity scores were additionally included as covariates to the models. All analyses were performed with IBM SPSS Statistics version 25.

## Results

### Study population

**Fig.1:**
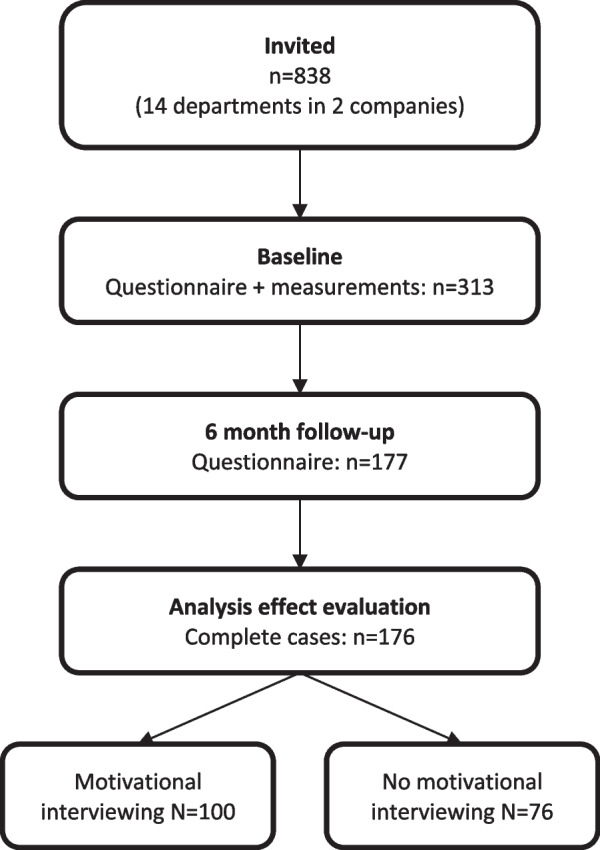
Flowchart of participants in the study

Figure 1 shows that 313 workers completed the PME and met the inclusion criteria for participation in the WHPP. Of these 313 workers, 177 workers also responded to the 6 month follow-up measurements (56.5%), with 176 complete cases. Among these 176 workers who both completed the PME and filled out the 6-month questionnaire, 100 workers attended at least 1 MI-coaching session and 76 workers did not participate in MI coaching. The majority of the participants were men, and had an intermediate education level (Table 1). About 40% of the participants were at least 50 years old. The study population mainly consisted of shift workers at the production company, who worked at least 36 h per week. Most workers (77%) experienced good or very good health at baseline, 61% did not smoke, and 78% consumed 7 glasses of alcohol or less per week. The majority of workers (68%) had overweight or obesity, and did not meet the Dutch guidelines on fruit- (56%) and vegetable (84%) consumption. In total, 79% of the workers who attended MI coaching did not meet the recommendations for vigorous physical activity at baseline, compared to 46% of the workers without MI coaching. Most participants (89%) had a work ability score higher than 6 and 80% had less than 10 days of sickness absence. Loss to follow-up after 6 months was higher among workers who experienced financial pressure, workers with a healthy weight, heavy physical workload, and was lower among shift workers, those working at least 36 h per week, or those working at the production company Additional file 1.

**Table 1:** Characteristics of the study population participating in the preventive medical examination at baseline

	Motivational interviewing (*N* = 100)		No motivational interviewing (*N* = 76)	
	N	%	N	%
**Demographics**				
Gender male	91	91.0^a^	57	75.0
Age				
< 30 years	19	19.0	15	19.7
30–40 years	19	19.0	17	22.4
40–50 years	21	21.0	14	18.4
≥ 50 years	41	41.0	30	39.5
Education				
High	3	3.0	6	7.9
Intermediate	57	57.0	55	72.4
Low	40	40.0^a^	15	19.7
Financial pressure	10	10.0	9	11.8
Married/cohabiting	75	75.0	50	65.8
**Health and health behaviours**				
Good/very good self-rated health	73	73.0	63	82.9
Healthy weight	23	23.0^a^	33	43.4
≥ 2 days a week vigorous physical activity	21	21.0^a^	41	53.9
Non-smoker	56	56.0	51	67.1
≤ 7 glasses of alcohol a week	76	76.0	61	80.3
≥ 2 pieces of fruit a day	42	42.0	35	46.1
≥ 4 servings of vegetables a day	14	14.0	14	18.4
**Work-related factors**				
Work ability > 6	90	90.0	67	88.2
< 10 days sickness absence	76	76.0	65	85.5
**Working conditions**				
Working ≥ 36 h a week	94	94,0	70	92,1
Low autonomy	37	37.0	23	30.3
High work pressure	14	14.0	8	10.5
Heavy physical workload	36	36.0	23	30.3
Shift work	99	99.0^a^	71	93.4
Mainly physically strenuous tasks	7	7.0	6	7.9
Less than good work-life balance	15	15.0	9	11.8
Working in production company	99	99.0^a^	67	88.2

^a^Chi-square is statistically significant at the 0.05 level

### Characteristics associated with participation in the intervention

Of the workers with overweight and obesity at baseline, respectively 60% and 73% participated in MI coaching. The majority of smokers (64%), workers with a lack of vigorous physical activity (69%), high alcohol consumption (62%), and insufficient fruit (59%)- and vegetable consumption (58%) at baseline participated in subsequent MI coaching. Table 2 shows that male workers [odds ratio (OR) = 3.05, 95% confidence interval (CI): 1.06;8.81], those with a low education (OR = 3.54, 95%CI: 1.46;7.68), workers who were either overweight (OR = 2.98, 95%CI: 1.28;6.93) or obese (OR = 5.20, 95%CI: 1.87;14.46), and in particular those who did not meet the guidelines for vigorous physical activity (OR = 7.09, 95%CI: 3.22;15.64) and worked at the production company (OR = 12.39, 95%CI: 1.28;119.80) were more likely to participate in subsequent MI coaching after completing the PME compared to workers who did not attend the MI sessions. Together, these determinants explained between 29% (R^2^ according Cox & Snell) and 38% (Nagelkerke R^2^) of the variation in MI participation. For 81% of the workers, classification in the group with MI versus the group without MI was correctly done based on these determinants [Area Under the Curve (AUC) = 0.81, 95%CI: 0.74;0.87]. Therefore, the performance of the prediction model estimating the propensity score is considered as good. Additional file 2 shows the associations between all baseline characteristics and participation in MI after the PME, with each association independently tested.

**Table 2:** Characteristics contributing most to participation in the preventive medical examination and subsequent motivational interviewing

Characteristics	Motivational interviewing (*N* = 100)
	OR (95% CI)
Gender	
Female (ref.)	1
Male	**3.05** (1.06;8.81)
Education	
High or intermediate (ref.)	1
Low	**3.54** (1.53;8.20)
BMI	
Healthy weight (ref.)	1
Overweight	**2.98** (1.28;6.93)
Obese	**5.20** (1.87;14.46)
Vigorous physical activity	
2 days or more p/w (ref.)	1
< 2 days	**7.09** (3.22;15.64)
Organisation	
Hospital (ref.)	1
Production company	**12.39** (1.28;119.80)
R2 (range Cox & Snell to Nagelkerke)	(28.5%-38.2%)
Area Under the Curve (95%CI)	**0.81** (0.74;0.87)

Bold: estimate is significant at the 0.05 level

### Effectiveness of the intervention

Workers who participated in MI coaching sessions were almost 5 times (OR = 4.74, 95%CI: 1.99;11.32) more likely to participate in health promotion activities after the PME in the fully adjusted model. As presented in Table 3, BMI and bodyweight decreased after 6 months for both workers who attended MI sessions and those who did not participate in MI. Persons who participated in MI sessions had a lower decrease in BMI (b = 0.39, 95%CI: 0.02;0.76) and bodyweight (b = 1.19, 95%CI: 0.02;2.36) compared to those without MI. These associations reduced slightly and became non-significant after adjusting for the propensity score. Participation in MI sessions was associated with 16.7% (95%CI: 1.5;31.8) more workers who started to perform sufficient vigorous physical activity after 6 months compared to workers who did not participate in MI sessions. This association reduced slightly and became non-significant when accounting for the propensity score. In total, 10.9% (95%CI: 0.6;21.3) more persons quit smoking in the intervention group compared to the reference group in the fully adjusted model. Although the proportions of persons with low alcohol intake increased only among workers without MI, and the prevalence of workers with sufficient fruit intake decreased after 6 months in both groups, no statistically significant effects of MI coaching were found for these outcomes and other outcomes related to health behaviour and work.

**Table 3:** Effectiveness of motivational interviewing in improving health, health behaviour, work ability and sickness absence

	Motivational interviewing (*N* = 100)		No motivational interviewing (*N* = 76)		Difference in 6 month change between intervention and reference	
	Baseline	6 months	Baseline	6 months	Model 1^a^	Model 2^b^
**Continuous outcome measures**	Mean (SD)	Mean (SD)	Mean (SD)	Mean (SD)	B (95% CI)	B (95% CI)
BMI (kg/m^2^)	28.15 (4.68)	27.81 (4.25)^c^	26.25 (3.78)	25.82 (3.72)^c^	**0.39** (0.02;0.76)	0.32 (-0.09:0.73)
Bodyweight (kg)	89.24 (15.70)	88.15 (14.32)^c^	82.11 (15.27)	80.75 (15.06)^c^	**1.19** (0.02;2.36)	1.04 (-0.27;2.34)
Work ability (0–10)	8.09 (1.36)	7.91 (1.44)	8.13 (1.26)	7.86 (1.47)	0.10 (-0.33;0.53)	0.07 (-0.42;0.56)
**Dichotomous outcome measures**	N (%)	N (%)	N (%)	N (%)	% (95% CI)	% (95% CI)
Good/very good self-rated health	73 (73.0)	77 (77.0)	63 (82.9)	60 (78.9)	1.8 (-9.9;13.6)	5.7 (-7.5;18.9)
≥ 2 days a week vigorous physical activity	21 (21.0)	62 (62.0)^c^	41 (53.9)	47 (61.8)	**16.7** (1.5;31.8)	15.8 (-0.1;31.8)
Non-smoker	56 (56.0)	67 (67.0)^c^	51 (67.1)	52 (68.4)	8.8 (-0.4;18.1)	**10.9** (0.6;21.3)
≤ 7 glasses of alcohol a week	76 (76.0)	84 (84.0)	61 (80.3)	67 (88.2)^c^	-2.6 (-11.7;6.4)	-3.5 (-13.8;6.8)
≥ 2 pieces of fruit a day	42 (42.0)	30 (30.0)^c^	35 (46.1)	23 (30.3)^c^	2.6 (-10.9;16.1)	4.4 (-10.8;19.7)
≥ 4 servings of vegetables a day	14 (14.0)	9 (9.0)	14 (18.4)	12 (15.8)	-4.4 (-14.7;5.8)	0.5 (-11.0;11.9)
< 10 days sickness absence	76 (76.0)	84 (84.0)	65 (85.5)	62 (81.6)	6.2 (-5.4;17.7)	10.0 (-3.1;23.1)

Bold: estimate is significant at the 0.05 level

*SD* standard deviation

^a^Associations are adjusted for baseline values, sociodemographic characteristics (gender, age and education), and organisation

^b^Model 1 + adjusted for propensity score

^c^Significant difference between baseline and 6 months at the 0.05 level

### Delivery of the intervention

Information from reports by 7 coaches on the number of MI coaching sessions and the duration of these sessions was available for 70 of the 100 workers who attended MI coaching. The majority of these workers attended 1 MI session (73%), 17% received 2 MI coaching sessions, and a small proportion (10%) participated in 3 or more sessions. The mean duration for each session was 31 min (SD = 10.5 min). Workers indicated in the follow-up questionnaire to have participated in on average 2 health promotion activities (SD = 1.3). The analysis on the quality of MI coaching was based on 23 audio recordings, with a mean duration of 15 min (SD = 6.8 min) per session. Audio recordings were not available for one coach. The number of recordings ranged between 1 and 7 for each professional. The mean technical- and relational scores were both 3.5 (SD = 0.5). The technical score is indicative of acceptable quality (> 3), and the relational score is considered as low quality (< 4) [22]. In addition, whereas the majority of the recorded professionals (83%) met the guideline of at least 40% complex reflections, 30% had an adequate ratio of reflections versus questions.

Table 4 shows that the vast majority of workers who attended MI coaching (strongly) agreed that the OP or other coaches treated them pleasantly, that the coaches had sufficient expertise, and that the contact with the coach lived up to their expectations. Workers were satisfied with the contact with coaches in general as well as with the number of contact moments. Most workers believed they gained knowledge through the coaches on how to improve health behaviour and that the contact contributed to change in health behaviour.

**Table 4:** The percentage of workers (strongly) agreeing with statements on the contact with coaches

% (Strongly) agreeing with statements on contact with coach	Occupational physician	Other coaches
Pleasant treatment by coach	92.7%	100%
Expertise of coach	95.1%	97.1%
Confidential treatment of medical data by coach	95.1%	100%
Satisfied with contact with coach	92.7%	100%
Contact with coach lived up to expectations	86.6%	88.6%
Knowledge gained on how to improve health behaviour through contact with coach	75.6%	91.4%
Contact with coach contributed to change in health behaviour	51.2%	82.9%
Satisfied with the number of contacts with coach	87.8%	94.3%

## Discussion

The current study showed that after participation in a PME the majority of the blue-collar workers with lifestyle-related risk factors were reached to participate in additional MI coaching sessions focused on self-motivated health behaviour change. The programme succeeded to reach workers with a low educational level to participate in additional MI coaching after the PME. Workers who attended MI coaching participated more often in additional health promotion activities and quit smoking more often after 6 months compared to those without MI coaching. Changes after 6 months in BMI, bodyweight, health, health behaviour, work ability, and sickness absence were not different for workers with and without subsequent MI coaching after the PME.

The findings suggest that the PME and subsequent MI coaching reached unhealthy workers, with unhealthy behaviour and those with low educational level. Previous research did not demonstrate consistent effects of health, health behaviour, and socioeconomic position on participation in WHPPs [6, 26]. By targeting workers in lower socioeconomic position and giving workers with an elevated health risk the opportunity to attend coaching with MI the current program reached, as intended, those for whom the greatest health gain could be achieved. The personalized results from the PME may have increased the perceived susceptibility of a health threat among workers with an elevated health risk [27], and therefore also the need to attend coaching. The relative higher participation of low educated workers in MI coaching after the PME could partly be attributed to the higher prevalence of obesity and unhealthy behaviours in this group [6], which determined whether they had the opportunity to receive MI coaching. Some evidence suggests a preference of individuals in low socioeconomic position for individually tailored counselling [28], which could also explain the higher likelihood for low educated workers to participate in MI coaching. However, other findings indicate that individually tailored interventions might be more suitable for workers in high socioeconomic position [29] and that workers with low socioeconomic position might prefer group based advice over individual advice [28].

This study showed that MI coaching in addition to the PME increased participation in health promotion activities and smoking quit rates after 6 months. This indicates that MI increases self-motivated behavioural change when complemented to health checks. In line with these findings, Groeneveld et al. [16] found in a randomized controlled trial (RCT) among male construction workers that smoking cessation after 6 months was higher for workers who received individual counselling with MI after a health screening compared to those who only attended the health screening. The effect size for smoking cessation in the current study was slightly lower (Cohen’s d = 0.23) compared to the study by Groeneveld et al. (Cohen’s d = 0.29), but slightly higher compared to individual counselling programs (Cohen’s d = 0.16) as reported by a Cochrane review on smoking cessation in the workplace [30]. These results indicate that the MI technique among blue-collar workers is beneficial for smoking cessation. An alternative explanation for the slightly lower effect size for smoking cessation found in the Cochrane review is the inclusion of studies with longer follow-up periods (6–24 months), which may have resulted in weakening of the intervention effect. Initially we intended to also evaluate the current program after 12 months, however this was not possible due to Covid-19 regulations.

According to the results in this study, the decrease in BMI was not different for workers who attended MI coaching than for those who only participated in the PME. Therefore, MI coaching was not sufficient to promote further change in BMI. Insufficient delivery of MI, regarding both quantity and quality, may have limited the beneficial impact on BMI. The majority of workers who attended coaching, participated in 1 session with a mean duration of 31 min. A possible explanation for this is that many follow-up sessions were cancelled due to Covid-19 regulations. Although results from a systematic review indicated that 1 MI session was effective in promoting change in health-related outcomes in primary care populations [31], multiple MI sessions with a longer duration may further increase readiness to change and participation in health promotion activities [32]. Concerning the quality of MI provided by coaches, the process evaluation showed a low relational quality score, in particular for partnership. This implies that coaches frequently took the expert role in the interaction with the worker (low partnership) instead of actively stimulating power sharing and the contribution of the worker (high partnership), which is assumed to discourage change [13]. This could also explain why the majority only participated in 1 MI session. Two or three training days may not be sufficient to avoid the so called ‘righting reflex’, which is the tendency of health professionals to inform or advise clients about health behaviour change in a directive way [13]. Moreover, the MI sessions took place several months (3–5 months) after the MI training. For future WHPPs, a shorter time between the MI training and the MI sessions and frequent intervision between coaches to discuss experiences might contribute to mastering MI skills. In addition, in line with a recent meta-analysis on Dutch WHPPs [33], the lack of a beneficial effect on BMI may also be explained by not addressing the underlying structural factors (e.g. community norms, physical constraints) that may prevent persons in low socioeconomic position to achieve good health. Strategies that include structural factors are more effective in preventing obesity among low socioeconomic groups [34]. Therefore, future workplace health promotion programs could incorporate a structural approach combined with MI coaching at the individual level to further decrease socioeconomic inequalities among workers in BMI.

This study has several strengths. The main strength is the evaluation of the WHPP in a real-life setting, since the coaching was provided by the professionals working within the organizations. This increases the generalizability to other organisations with workers in lower socioeconomic position. Other important strengths include the high participation in subsequent MI coaching of low educated workers with elevated health risks and the investigation of MI coaching in addition to a PME on a variety of outcomes related to health, health behaviour and work. Therefore, this study provided knowledge on MI as a promising method to increase reach and effectiveness among workers in lower socioeconomic position when complemented to assessment of workers’ health risks. Several limitations can also be addressed. The first limitation is the low study sample size. A lack of statistical power might explain why we found beneficial, but not statistically significant, effects of subsequent MI coaching following the PME on self-rated health, vigorous physical activity and sickness absence. Participation at baseline and after 6 months was especially low for workers in the hospital. In contrast to the production company, PMEs were not prioritized in the hospital before implementing the program. Moreover, in the hospital, workers were invited and reminded to take part in the program through email and both the PME and follow-up questionnaires were administered online instead of face-to-face. Since eHealth literacy is found to be lower among workers with a lower educational level [35] and active organizational support is found to increase participation in WHPPs [36], this might explain the low participation level in the hospital. Therefore, an existing PME procedure and possibilities to make the program more accessible and comprehensible for the target population may contribute to successful implementation of the program among workers in lower socioeconomic positions with varying occupations. Another possible explanation for the low participation levels in the hospital may be that workers experienced a major reorganization prior to our program. This resulted in some dissatisfaction among workers, problems adjusting to new circumstances, and consequently a lower willingness to participate. The second limitation is related to propensity score adjustment in the analyses. While the propensity score adjusted for observed characteristics which determined the likelihood to participate in subsequent MI coaching, it did not balance the unobserved characteristics between workers with and without MI coaching. However, propensity score adjustment is considered as a robust alternative method in occupational contexts where randomization is often not feasible [37]. The third limitation is that BMI was measured using self-reported data on bodyweight and height. This may have resulted in a lower prevalence of persons with overweight or obesity.

## Conclusions

Coaching with MI in addition to a PME provided to blue-collar workers reached, as intended, mainly workers in low education with elevated health risks, and increased participation in health promotion activities and smoking cessation after 6 months. The intervention however did not show effects on BMI, health behaviour, work ability and sickness absence. For future research it would be relevant to focus on increasing participation of workers in low socioeconomic position to PMEs and on sustainability of behaviour change among this group of workers by optimising the quantity and quality of MI, by addressing structural factors, and by evaluating the effectiveness over a longer follow-up period.

### Supplementary Information

**Additional file 1: Table S1.** Characteristics associated with loss to follow-up between baseline and 6 months.

**Additional file 2: Table S2.** Characteristics associated with participation in the preventive medical examination and subsequent motivational interviewing sessions (*N*=100) compared to not participating in coaching with motivational interviewing (*N*=76), with each association independently tested.

## Data Availability

The datasets used and/or analysed during the current study are available from the corresponding author on reasonable request.

## Abbreviations

BMI: Body mass index
WHPP: Workplace health promotion program
MI: Motivational interviewing
PME: Preventive medical examination
MITI: Motivational interviewing treatment integrity code
OP: Occupational physician
CI: Confidence interval
OR: Odds ratio
SD: Standard deviation
