# Supplementary material for: Reach and effectiveness of a worksite health promotion program combining a preventive medical examination with motivational interviewing; a quasi-experimental study among workers in low socioeconomic position
Source: BMC Public Health. 2023 Oct 31;23:2130. doi: 10.1186/s12889-023-16908-w (PMC10617210; doi:10.1186/s12889-023-16908-w)
Supplement: Supplementary file 1 — Additional file 1: Table S1. Characteristics associated with loss to follow-up between baseline and 6 months. [file 12889_2023_16908_MOESM1_ESM.docx]

**Reach and effectiveness of a worksite health promotion program combining a preventive medical examination with motivational interviewing; a quasi-experimental study among workers in low socioeconomic position**

**Additional file 1**

**Table S1.** Characteristics associated with loss to follow-up between baseline and 6 months.

|  | Non-response at follow-up |
| --- | --- |
|  | OR (95% CI) |
| **Demographics** |  |
| Gender male | 0.67 (0.38;1.20) |
| Age |  |
| 30-40 years | 0.94 (0.47;1.89) |
| 40-50 years | 0.74 (0.36;1.53) |
| ≥50 years | 0.94 0.51;1.72) |
| Education |  |
| Intermediate | 0.56 (0.22;1.40) |
| Low | 0.74 (0.28;1.94) |
| Financial pressure | **2.22** (1.18;4.16) |
| Married/cohabiting | 0.78 (0.48;1.26) |
| **Health and health behaviours** |  |
| Good/very good self-rated health | 0.77 (0.46;1.28) |
| Healthy weight | **1.88** (1.18;2.98) |
| ≥2 days a week vigorous physical activity | 1.27 (0.80;2.01) |
| Non-smoker | 0.93 (0.59;1.47) |
| ≤ 7 glasses of alcohol a week | 1.06 (0.62;1.82) |
| ≥2 pieces of fruit a day | 0.86 (0.55;1.36) |
| ≥4 servings of vegetables a day | 1.18 (0.65;2.13) |
| **Work-related factors** |  |
| Work ability >6 | 1.54 (0.69;3.42) |
| <10 days sickness absence | 0.78 (0.46;1.34) |
| **Working conditions** |  |
| Working ≥36 hours a week | **0.33** (0.16;0.68) |
| Low autonomy | 0.95 (0.59;1.52) |
| High work pressure | 0.93 (0.47;1.84) |
| Heavy physical workload | **1.69** (1.07;2.67) |
| Shift work | **0.27** (0.10;0.70) |
| Mainly physically strenuous tasks | 1.20 (0.53;2.73) |
| Less than good work-life balance | 1.78 (0.98;3.21) |
| Working in production company | **0.21** (0.10;0.46) |

Bold: estimate is significant at the 0.05 level
