# Supplementary material for: Reach and effectiveness of a worksite health promotion program combining a preventive medical examination with motivational interviewing; a quasi-experimental study among workers in low socioeconomic position
Source: BMC Public Health. 2023 Oct 31;23:2130. doi: 10.1186/s12889-023-16908-w (PMC10617210; doi:10.1186/s12889-023-16908-w)
Supplement: Supplementary file 2 — Additional file 2: Table S2. Characteristics associated with participation in the preventive medical examination and subsequent motivational interviewing sessions (N=100) compared to not participating in coaching with motivational interviewing (N=76), with each association independently tested. [file 12889_2023_16908_MOESM2_ESM.docx]

**Reach and effectiveness of a worksite health promotion program combining a preventive medical examination with motivational interviewing; a quasi-experimental study among workers in low socioeconomic position**

**Additional file 2**

**Table S2.** Characteristics associated with participation in the preventive medical examination and subsequent motivational interviewing sessions (N=100) compared to not participating in coaching with motivational interviewing (N=76), with each association independently tested.

|  | Motivational interviewing (N=100) |
| --- | --- |
|  | OR (95% CI) |
| **Demographics** |  |
| Gender male | **3.37** (1.43;7.96) |
| Age |  |
| 30-40 years | 0.88 (0.34;2.26) |
| 40-50 years | 1.18 (0.45;3.08) |
| ≥50 years | 1.08 (0.47;2.46) |
| Education |  |
| Intermediate | 2.07 (0.49;8.70) |
| Low | **5.33** (1.18;24.08) |
| Financial pressure | 0.83 (0.32;2.15) |
| Married/cohabiting | 1.56 (0.81;3.00) |
| **Health and health behaviours** |  |
| Good/very good self-rated health | 0.56 (0.27;1.17) |
| Healthy weight | **0.39** (0.20;0.75) |
| ≥2 days a week vigorous physical activity | **0.23** (0.12;0.44) |
| Non-smoker | 0.62 (0.34;1.16) |
| ≤ 7 glasses of alcohol a week | 0.78 (0.38;1.61) |
| ≥2 pieces of fruit a day | 0.85 (0.47;1.55) |
| ≥4 servings of vegetables a day | 0.72 (0.32;1.62) |
| **Work-related factors** |  |
| Work ability >6 | 1.21 (0.47;3.14) |
| <10 days sickness absence | 0.54 (0.24;1.18) |
| **Working conditions** |  |
| Working ≥36 hours a week | 1.34 (0.42;4.34) |
| Low autonomy | 1.35 (0.72;2.56) |
| High work pressure | 1.38 (0.55;3.49) |
| Heavy physical workload | 1.30 (0.69;2.45) |
| Shift work | 6.97 (0.80;60.97) |
| Mainly physically strenuous tasks | 0.88 (0.28;2.73) |
| Less than good work-life balance | 1.31 (0.54;3.19) |
| Working in a production company | **13.30** (1.65;107.42) |

Bold: estimate is significant at the 0.05 level
